# Effect of hypoxia factors gene silencing on ROS production and metabolic status of A375 malignant melanoma cells

**DOI:** 10.1038/s41598-021-89792-2

**Published:** 2021-05-14

**Authors:** Ivana Špaková, Miroslava Rabajdová, Helena Mičková, Wolfgang F. Graier, Mária Mareková

**Affiliations:** 1 Department of Medical and Clinical Biochemistry, Faculty of Medicine, Pavol Jozef Šafárik University in Košice, Trieda SNP 1, 04011 Košice, Slovakia; 2 Department of Biology, Faculty of Medicine, Pavol Jozef Šafárik University in Košice, Košice, Slovakia; 3 Gottfried Schatz Research Center for Cell Signaling, Metabolism and Aging Molecular Biology and Biochemistry, Medical University of Graz, Graz, Austria; 4 BioTechMed, Graz, Austria

**Keywords:** Biochemistry, RNA

## Abstract

The innate response of melanocytes to exogenous or endogenous stress stimuli like extreme pH and temperature, metabolite and oxygen deficiency or a high UV dose initiates a cellular stress response. This process activates adaptive processes to minimize the negative impact of the stressor on the pigment cell. Under physiological conditions, a non-cancer cell is directed to apoptosis if the stressor persists. However, malignant melanoma cells will survive persistent stress thanks to distinct "cancerous" signaling pathways (e.g. MEK) and transcription factors that regulate the expression of so-called "survival genes" (e.g. HIF, MITF). In this survival response of cancer cells, MEK pathway directs melanoma cells to deregulate mitochondrial metabolism, to accumulate reduced species (NADH), and to centralize metabolism in the cytosol. The aim of this work was to study the effect of gene silencing in malignant melanoma A375 cells on metabolic processes in cytosol and mitochondria. Gene silencing of HIF-1α, and miR-210 in normoxia and pseudohypoxia, and analysis of its effect on MITF-M, and PDHA1 expression. Detection of cytosolic NADH by Peredox-mCherry Assay. Detection of OCR, and ECAR using Seahorse XF96. Measurement of produced O_2_^•−^ with MitoTracker Red CMXRos. ^1^H NMR analysis of metabolites present in cell suspension, and medium. By gene silencing of HIF-1α and miR-210 the expression of PDHA1 was upregulated while that of MITF-M was downregulated, yielding acceleration of mitochondrial respiratory activity and thus elimination of ROS. Hence, we detected a significantly reduced A375 cell viability, an increase in alanine, inositol, nucleotides, and other metabolites that together define apoptosis. Based on the results of measurements of mitochondrial resipiratory activity, ROS production, and changes in the metabolites obtained in cells under the observed conditions, we concluded that silencing of HIF-1α and miR-210 yields apoptosis and, ultimately, apoptotic cell death in A375 melanoma cells.

## Introduction

Over the last two decades the scientific community has been focused on the hypoxic microenvironment of tumours. Hypoxia-inducible factor signalling pathway (HIF-signalling) is described for different tissues such as brain^1,2^, lungs^3,4^, cardio-vascular system^5,6^, muscles^7–9^ or skin^10–14^. In the given tissues, HIF-signalling has a different priority compared to the rest of the affected growth, metabolic and transcription pathways^15–18^. In the case of malignant melanoma (MM) the HIF-signalling pathway is one of the most dominant pathways^11,19,20^. Malignant melanoma is an oncological skin disease, the presentation of which increases in populations living in temperate climate areas and is connected to pigmentation as well as thicknesses of the skin, high dose of UV radiation and positive family history for MM^21–25^. Malignant melanoma is one of the most aggressive malignancies^26^. The epidemiology of malignant melanoma in Europe is based on three main factors; age (over 40 y), male gender, and location (head/neck, trunk, upper and lower extremity)^27–30^. The incidence of MM in Europe is increasing at a rate of 3–7%, 144,000 new cases per year, and 27,000 deaths per year^31^. This disease has relatively fast slope because it often takes only six to nine months from late diagnosis to patient’s death^32^.

Hypoxia-inducibile factor 1α signaling pathway (HIF-1α) is hyperactivated in long-term anoxia conditions^33^ and even at physiological O_2_ concentration and induces pseudohypoxic conditions in the cell, increases oxidative stress, and ROS production. Due to BRAF (V-raf murine sarcoma viral oncogenes homolog B1) mutations cells increase activation of MEK pathway (p38/p38y) in an effort to ensure sufficient oxygen supply (O_2_). A major activator of MAPK/ERK kinase, the so-called MEK (MAPK—Mitogen-activated protein kinase; ERK—Extracellular-signal-regulated kinase), is BRAF. The mutation of the BRAF gene, referred to as BRAF^V600E^, is 90% of the T to A switch at the 1799th position of exon 15, replacement of the amino acid valine with glutamic acid at position 600 in the protein residue^34^. The BRAF activating mutations occur in about 50% of the cutaneous melanomas with BRAF^V600E^ being the most frequent variant^35^. Furthermore, the activity of transcription factors (eg. MITF—Microphthalmia-associated transcription factor; a regulator of melanocyte development and a key melanogenesis factor) that are capable of independently producing growth and regulatory signals (TFEB and TFE3 regulating lysosomal activity and autophagy) is increased^34,36^ and gather up the energy metabolism in the cytosol (glycolysis)^37^. Under physiological conditions the MAPK pathway decreases MITF activity by ubiquitin-dependent proteolysis. However, due to reciprocal p38 phosphorylation the MEK cascade can promote MITF expression leading to melanocyte differentiation and onco-melanogenesis^38^. Overexpression of MITF is associated with a negative progression of melanoma disease. Increased MITF gene amplification was detected in 10% of primary cutaneous melanoma and 15–20% of metastatic melanomas^39^.

Our aim was to study metabolic (glycolysis, mitochondrial viability, metabolite accumulation) and genetic effects (MITF-M, PDHA1 expression) due to hypoxa-miR miR-210, and HIF-1α silencing. Our measurements support the theory of pro-apoptotic signal induction in targeted silencing of hypoxic factors. The present study suggests that by attenuating the action of hypoxamir miR-210 or HIF-1α, mitochondrial respiration acceleration is achieved, thereby releasing ROS and subsequently labeling the cell for self-destruction. Thus, a more thorough investigation of mitochondrial activity in MM cells can be a key for development of organelle targeting therapy.

### Formation of ROS by pigment cells

**Figure 1:**
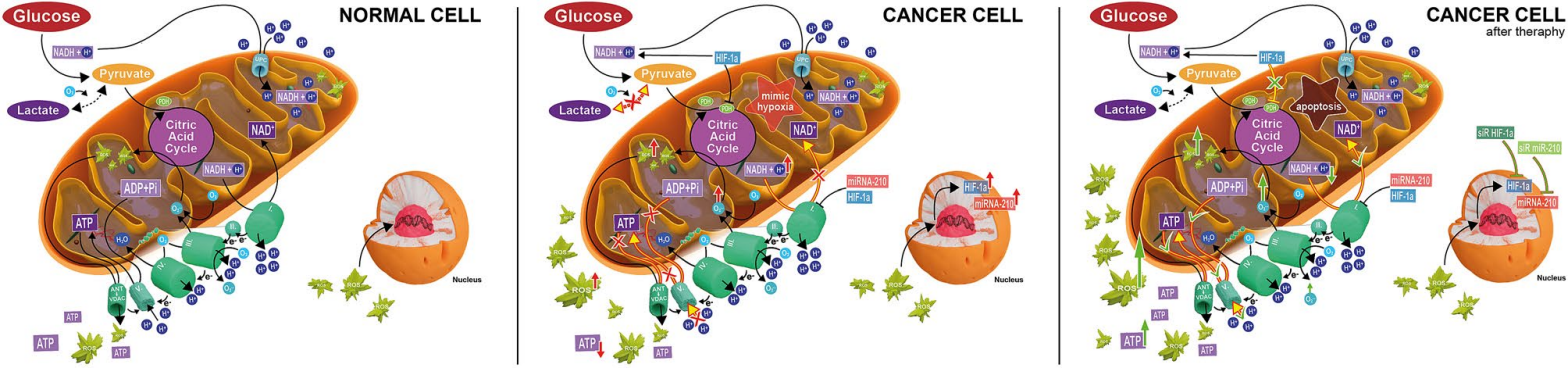
Mitochondria; normal cell: ROS is produced naturally, this ROS are deactivated by the anti-oxidation activity of enzymes as SOD which expression is stimulated; cancer cell: due mimic hypoxia cells uncouple the OXPHOS, accumulate NADH, increase ROS and activate expression of hypoxic survival genes as HIF-1α and miR-210; cancer cell after gene-therapy: due to gene silencing of HIF-1α and miR-210, the cancer cells lead to apoptosis because of re-activating of OXPHOS, increase in ROS and secreting of apoptic molecules.

It is well known that mitochondria (especially respiratory complex I in inner membrane) play an irreplaceable role in cell proliferation, calcium signaling modulation, and ROS production which serve as signaling molecules in downstream cell cycle control pathways such as apoptosis^40^. Isolated complex I deficiency is a very common cause of mitochondrial disorders that lead to a wide range of diseases^41^. Mitochondrial disorders are associated with mutations in the genes encoding complex I subunits and repair factors are in turn associated with an increase in ROS (Fig. 1)^42^.

Cells with insufficient respiratory chain activity are well resistant to mitochondrial stress and endoplasmic reticulum (ER) stress. In contrast, cells with active respiratory chain complexes but without the ability to generate electron flux are resistant to mitochondrial stress but sensitive to ER stress. Cells with partial electron flux reduction are sensitive to apoptotic signals from both organelles^42^. The resistance of cancer cells to apoptotic signals is attributed to the increased concentration of substrate NADH for the Complex I due to respiratory complex dysfunction^43,44^.

Production of ROS (H_2_O_2_, O_2_^•−^, and others) in subcellular structures is mainly located in microsomes (45%), peroxisomes (35%), mitochondria (15%), and in cytosol by specific enzymes (5%)^45^. The production of ROS in cell substructures is stable under physiological conditions. Approximately half of the total amount of hydrogen peroxide produced in mitochondria is generated by respiratory complexes I and III, whereas complex I generates up to 70% of ROS^45^. The second half of the total amount of ROS produced by mitochondria comes from metabolic processes of enzymes dissolved in the mitochondrial matrix, mainly dihydrolipoamide dehydrogenase (DLDH) and from submitochondrial particles (SMP)^45^.

In an attempt to compensate for the excessive accumulation of NADH, mitochondria utilize the present O_2_ in complex I to re-oxidize NADH, which leads to the formation of NAD^+^, H^+^ and O_2_^•−^ followed by H_2_O_2_^42^. According to one of the theories, complex I is inhibited by high NADH concentration suggesting that NADH can bind to an unconventional allosteric site changing the redox potential and O_2_ availability^45^. As a result, hydrogen peroxide formed by complex III accumulates in the mitochondrial matrix, intermembrane space (IMS), and cytosol of cancer cells^45–47^.

### Energy metabolism of melanoma cells

The activity of OXPHOS (oxidative phosphorylation) is dependent on NADH + H^+^ and FADH_2_ produced in the citrate cycle, the activity of which is controlled by the pyruvate dehydrogenase complex (PDC—pyruvate dehydroganase, dihydrolipoamide acetyltransferase and dihydrolipoamide dehydrogenase)^48^. The oxidative decarboxylation of pyruvate to Acetyl-CoA, CO_2_, and NADH catalyzed by PDC links glycolysis in the cytosol to the citrate cycle cell in mitochondria. Aberrant HIF-1α expression affects the disconnection of OXPHOS from energy generation, and cells preferentially utilize glycolysis. Malignant melanoma (MM) tumour cells are described as glycolytic hypoxic cells^49^ and overexpress HIF-1α in an attempt to de novo vascularization (upregulation of VEGF) and thereby compensate for O_2_ deficiency^50^.

Cells suffering from hypoxia (pseudohypoxia) due to decreased activity of respiratory complex I and/or due to mutation of the NADH-transdehydrogenase subunit leads to accumulation of NADH (NADPH + NAD^+^  ↔ NADP^+^  + NADH) which slows down and stops the citrate cycle and thus disconnects OXPHOS^51^. In melanoma cells, ROS production increases due to inactive OXPHOS^52,53^, whereas the lack of O_2_ associated with reduced NADH transformation into NAD^+^ leads to pseudohypoxia^45^. These are contradictory events resulting from damage to the respiratory chain complex I^51^. Complex I mutations inactivating NADH-ubiquinone subunits and other molecules of O_2_ responsible for O_2_^•−^ generation may be so destructive that cells reduce ROS production by the complex I^54^. Complex I mutations may therefore act as an accelerator of tumour progression due to ROS release, as well as negatively affect disease progression due to complex I disintegration.

Hypoxa-miR miR-210 stabilizes HIF-1α by inactivating the HIF-1α-inhibitor GPD1L (glycerol-3-phosphate dehydrogenase 1-like)^55,56^. miR-210 negatively affects the activity of ISCU electron transporters in respiratory complex I and is thus largely involved in the inactivation of OXPHOS, and subsequent accumulation of NADH and ROS in the mitochondrial matrix and IMS^57,58^. HIF-1α, as a major regulator of adaptation to hypoxic conditions, alters the expression of PGC-1α (peroxisome proliferator-activated receptor-γ coactivator 1α) to stimulate mitochondrial biogenesis^59^. Induction of HIF-1α-dependent genes on PGC-1α stimulus under normoxic conditions is a cell response to stabilization of HIF-1α by miR-210^60^.

MITF adjusts the expression of 'OXPHOS genes' by directly influencing mitochondrial regulators (e.g. as PGC-1α) and MITF also influences PDH^48^ by negative regulation of expression of the genes encoding vacuolar H^+^-ATPase subunits (v-ATPase)^61^ which results in proton accumulation in IMS and the lowering of pH. MITF in lysosomal pH-homeostasis regulates melanin formation^62^ in melanosomes (lysosome-like organelles) and its distribution to keratinocytes. Defective melanin results in cancer melanogenesis in amelanotic cells along with an increase in HIF-1α expression^63^. Stress-induced autophagy (lack of nutrients, hypoxia, ROS, and others) regulated by MITF factors promotes proteosynthesis and lipogenesis for subsequent proteolysis and β-oxidation of fatty acids, thereby generating ATP by OXPHOS for melanoma cell survival^36^, and at the same time a high level of NADH inhibits this process resulting in the dominance of glycolysis as the main energy pathway of the melanoma cell^64^.

## Results

### Gene expression

By RT-PCR analysis we detected changes in the target gene expression. Cells affected with siR HIF-1α were manifested with 56% decrease in MITF-M and 39% decrease in PDHA1 compared to siR neg cont cells in normoxia and 33% decrease in MITF-M, and 4% increase in PDHA1 compared to siR neg cont cells in hypoxia. Cells affected with siR miR-210 were presented with 28% decrease in MITF-M, and 14% increase in PDHA1 compared to siR neg cont cells in normoxia and 59% decrease in MITF-M, and 8% increase in PDHA1 compared to siR neg cont cells in hypoxia.

**Figure 2:**
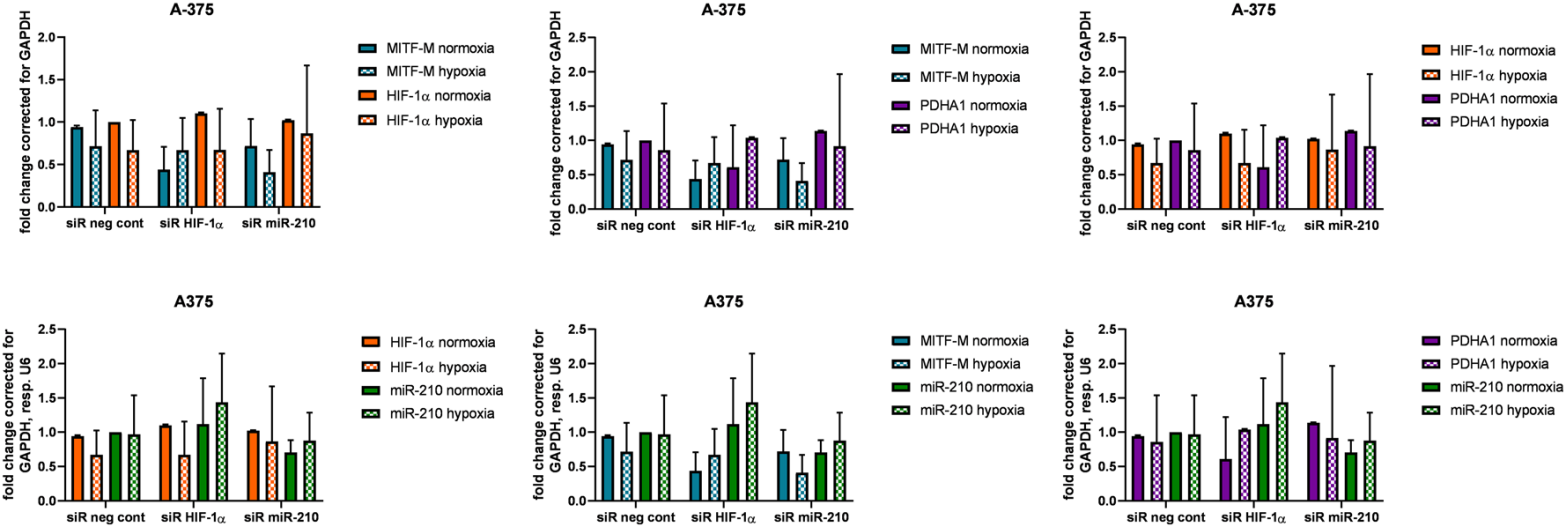
Relative gene expression of HIF-1α, miR-210, MITF-M, PDHA1 in normoxia and hypoxia of siR neg cont cells, siR HIF-1α, siR miR-210 (significant change with *P* value < 0.05 marked with *, *P* value < 0.01 marked with **, *P* value < 0.001 marked with ***). Relative expression values were analysed by REST Software (Qiagen).

The evaluation of gene expression in the relationship between experimental groups in normoxia and hypoxia, and in the relationship between individual genes in the experimental group is gathered in Table 1 and Fig. 2 (see more in supplemetary ST 2).

**Table 1:** Comparison of the relative expression values to siR neg cont in normoxia for siR HIF-1α and siR miR-210 and comparison of the relative expression values to siR neg cont in hypoxia for siR HIF-1α and siR miR-210 in hypoxia.

	Normoxia siR neg cont			Hypoxia siR neg cont	
	siR HIF-1α	siR miR-210	siR neg cont (hypoxia)	siR HIF-1α	siR miR-210
MITF-M	↓ 56%	↓ 28%	↓ 28%	↓ 33%	↓ 59%
PDHA1	↓ 39%	↑ 14%	↓ 14%	↑ 4%	↑ 8%
HIF-1α	↑ 10%	↓ 2%	↓ 33%	↓ 33%	↓ 13%
miR-210	↑ 12%	↓ 30%	↓ 3%	↓ 30%	↓ 59%

### Peredox-mCherry T-Sapphire Assay

**Figure 3:**
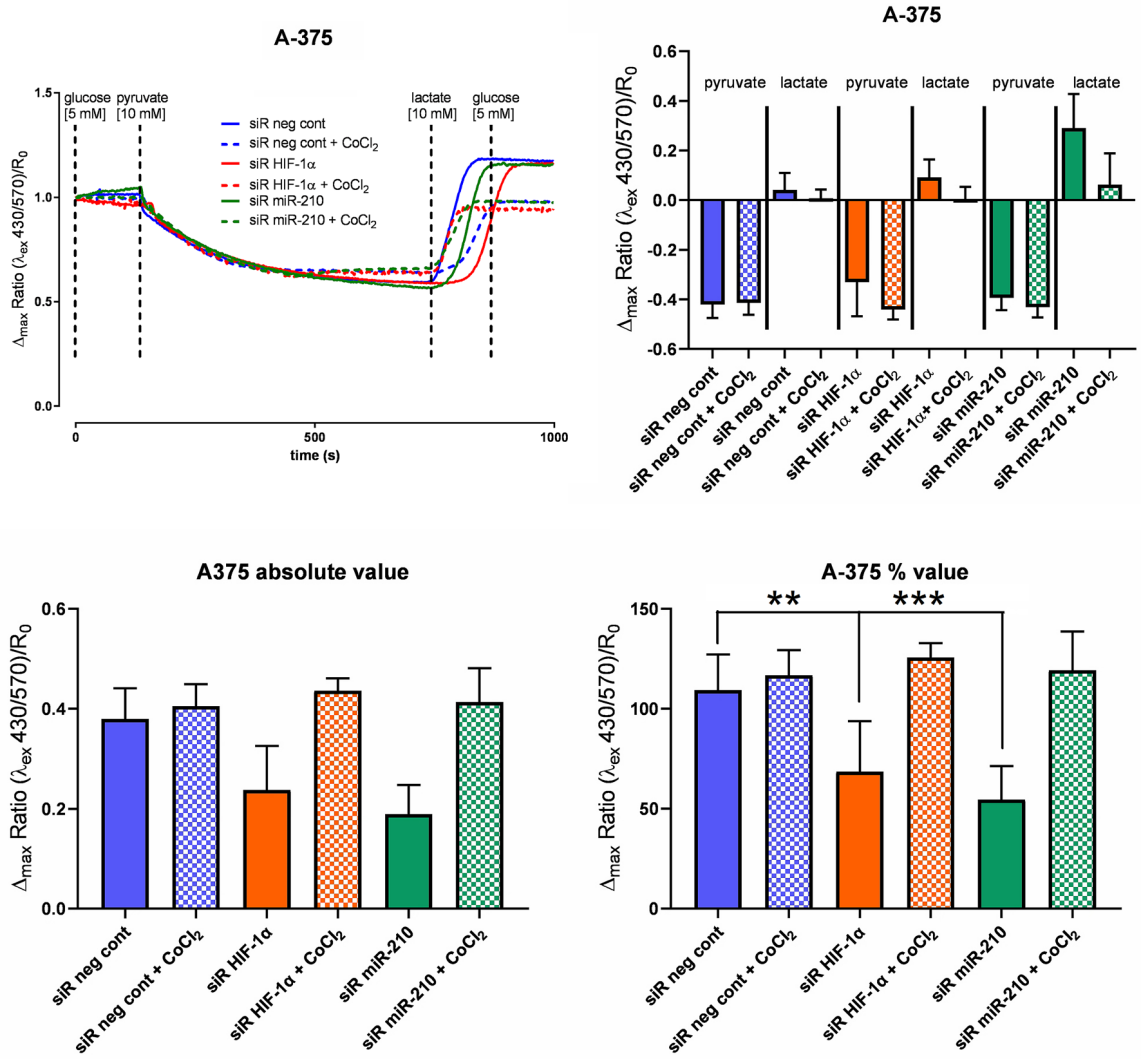
Cytosolic NADH/NAD^+^ ratio; the level of free NADH decreased in normoxia and slightly decreased in hypoxia (significant change with *P* value < 0.05 marked with *, *P* value < 0.01 marked with **, *P* value < 0.001 marked with ***).

The production of NADH by MM A375 cells under normoxic and pseudohypoxic conditions was approximately at the same level and a slight increase of ± 7% was observed in the control cells in induced hypoxia. We detected a significant reduction of the free NADH/NAD^+^ ratio in normoxia by ± 37% (*P* = 0.0031) after HIF-1α silencing, and in miR-210 by ± 50% (*P* < 0.0001). An increase in the free NADH/NAD^+^ ratio in pseudohypoxia after silencing of HIF-1α by ± 15% (*P* = 0.0439), and in miR-210 by ± 9% (*P* = 0.2501) was found compared to the control in normoxia (Fig. 3, supplementary data ST 3).

### Seahorse assay

**Figure 4:**
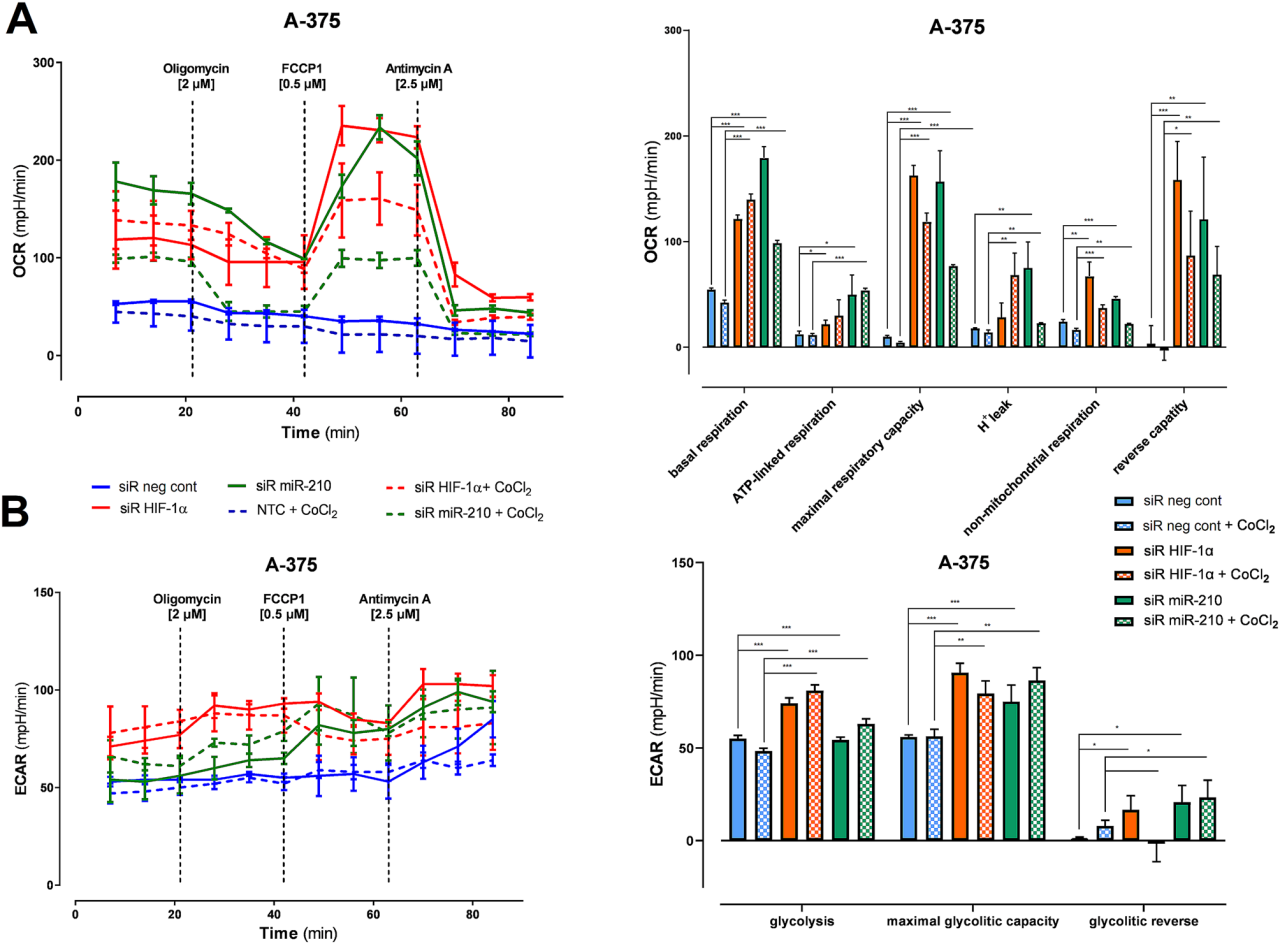
Oxygen consumption rate and extracellular acidification rate; mitochondria viability (basal respiration, ATP production, reverse capacity) of malignant melanoma cells was boosted after gene silencing (HIF-1α, miR-210) in normoxia as well as in hypoxia (significant change with *P* value < 0.05 marked with *, *P* value < 0.01 marked with **, *P* value < 0.001 marked with ***).

Determination of mitochondrial viability from OCR measurements according to Seahorse Assay recommendations^65^ (Fig. 4A, Table 2, supplementary data ST 4). From analysis of ECAR measurements according to the recommendations of the Seahorse Assay^65^ (Fig. 4B, Table 3, supplementary data ST 5).

**Table 2:** Comparison of the OCR values to siR neg cont in normoxia for siR HIF-1α and siR miR-210.

	Normoxia siR neg cont			Hypoxia siR neg cont	
	siR HIF-1α	siR miR-210	siR neg cont (hypoxia)	siR HIF-1α	siR miR-210
Basal respiration	↑ 111%	↑ 210%	↓ 25%	↑ 144%	↑ 81%
v-ATPase	↑ 76%	↑ 302%	↓ 5%	↑ 142%	↑ 335%
Maximal respiration capacity	↑ 1529%	↑ 1471%	↓ 55%	↑ 1090%	↑ 668%
H^+^ leak	↑ 58%	↑ 319%	↓ 21%	↑ 282%	↑ 27%
Non-mitochondrial respiration	↑ 176%	↑ 89%	↑ 16%	↑ 53%	↓ 9%
Reverse capacity	↑ 4427%	↑ 3364%	↓ 15%	↑ 2384%	↑1866%

Comparison of the OCR values to siR neg cont in hypoxia for siR HIF-1α and siR miR-210 in hypoxia.

**Table 3:** Comparison of the ECAR values to siR neg cont in normoxia for siR HIF-1α and siR miR-210.

	Normoxia siR neg cont			Hypoxia siR neg cont	
	siR HIF-1α	siR miR-210	siR neg cont (hypoxia)	siR HIF-1α	siR miR-210
Glycolysis	↑ 17%	↓ 14%	↓ 24%	↑ 28%	↓ 13%
Glycolytic capacity	↑ 5%	↓ 14%	↓ 35%	↑ 9%	↓ 36%
Glycolysis reserve	↑ 1566%	↑ 1966%	↓ 800%	↑ 66%	↑ 2233%

Comparison of the ECAR values to siR neg cont in hypoxia for siR HIF-1α and siR miR-210 in hypoxia.

### MitoTracker Red CMXRos assay

**Figure 5:**
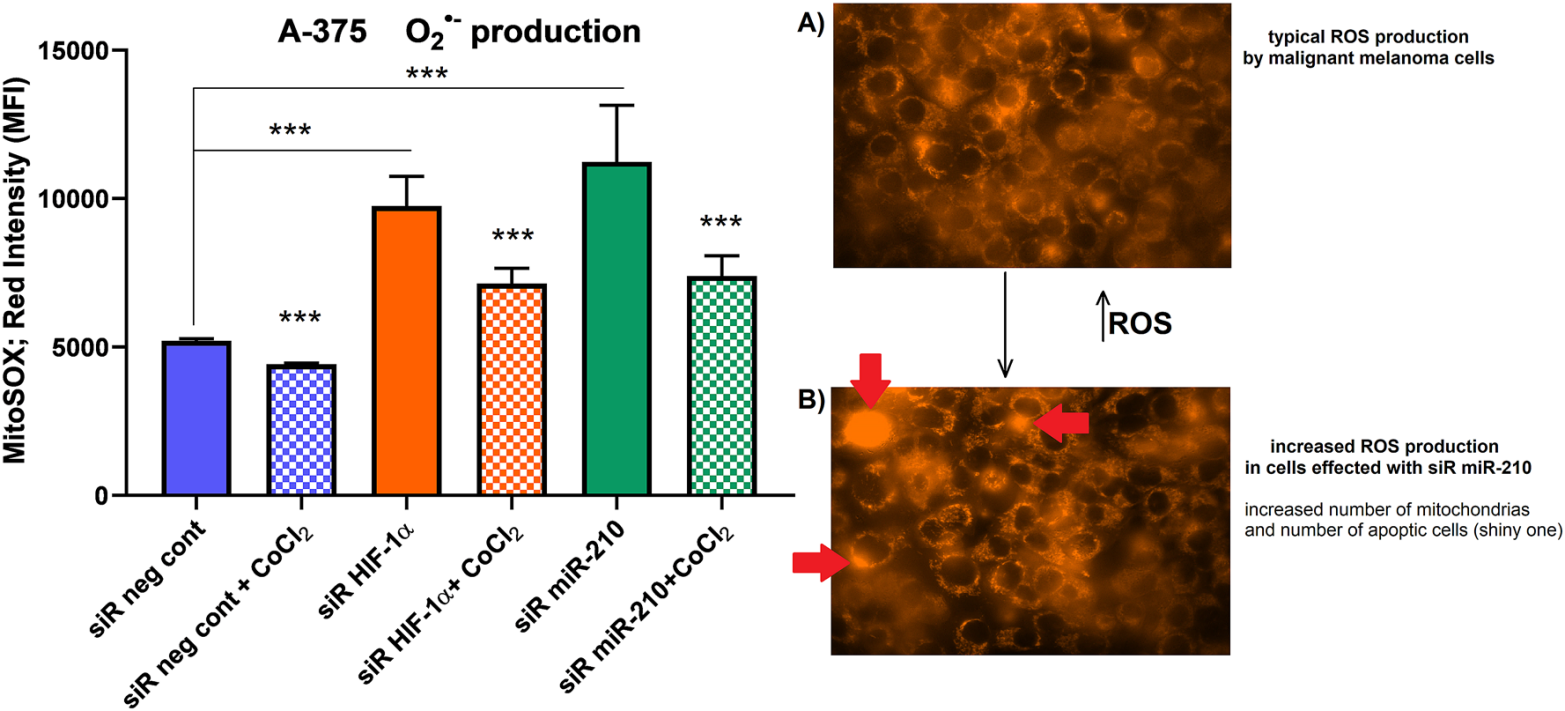
ROS production: box-plot describes changes in the ROS level in A375 cells in normoxia and hypoxia (blue: siR neg cont in normoxia, blue squared: siR neg cont in hypoxia, orange: gene silencing of HIF-1α in normoxia, orange squared: gene silencing of HIF-1α in hypoxia, green: gene silencing of miR-210 in normoxia, green squared: gene silencing of miR-210 in hypoxia). (**A**) A375 cells in hypoxia (intensity of the “red light” depends on ROS production); (**B**) A375 cells after gene silencing of miR-210 in hypoxia (red arrows target apoptic cells). Analysed by VisiView acquisition software (Universal Imaging, Visitron Systems).

In terms of O_2_^•−^ production and accumulation in mitochondria (Fig. 5, supplementary data ST 6), we observed a decrease in ROS molecules by ± 15% in hypoxia. After HIF-1α gene silencing the production of O_2_^•−^ increased by ± 87% in normoxia, and by ± 37% in hypoxia compared to normoxia control. We observed a more significant increase in ROS ± 115% after silencing of miR-210 in normoxia, and by ± 41% in hypoxia compared to the control group in normoxia.

### 1D ^1^H NMR

**Figure 6:**
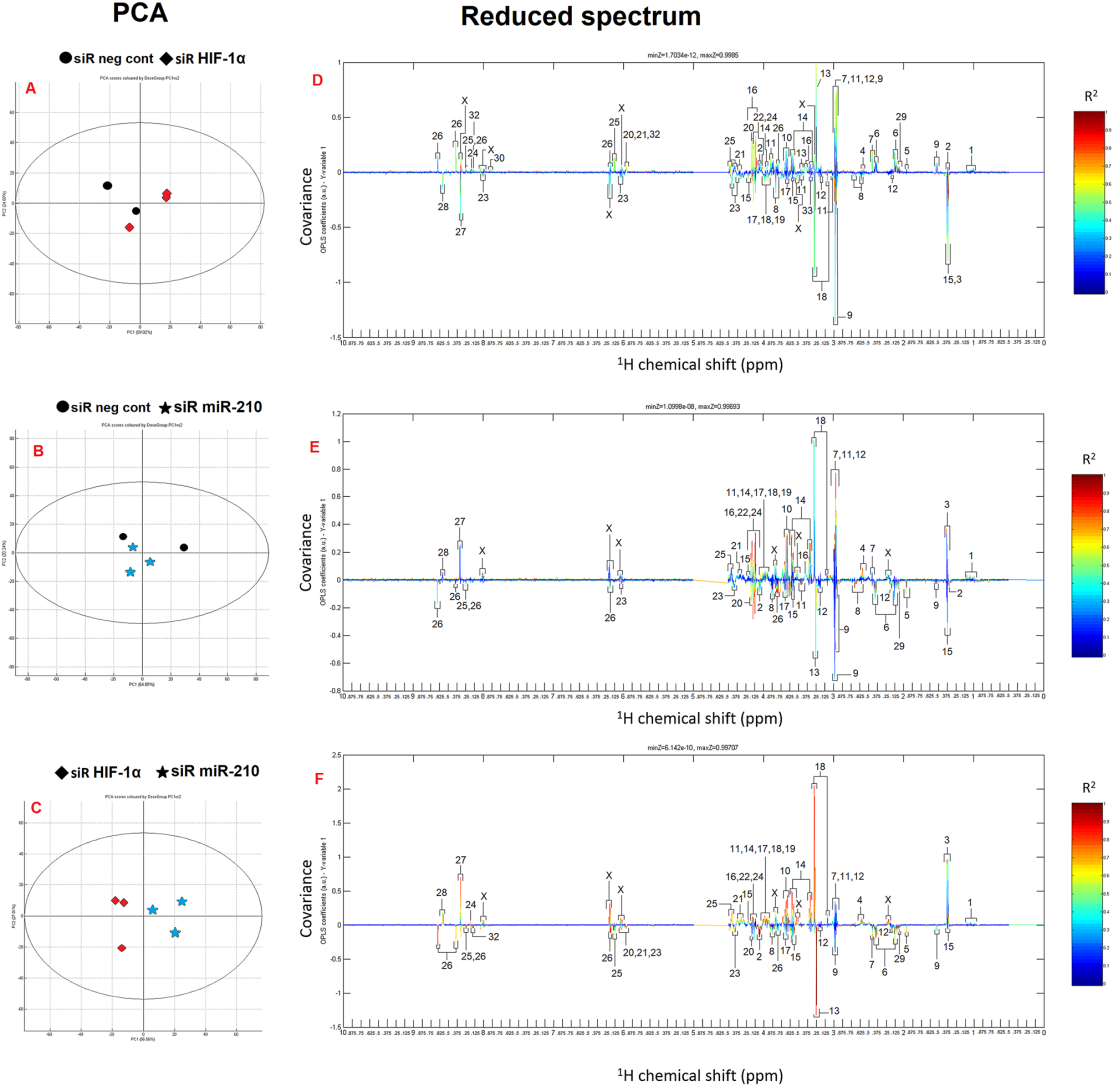
PCA model between the groups (**A**) siR neg cont and siR HIF-1α, (**B**) siR neg cont and siR miR-210, (**C**) siR HIF-1α and siR miR-210; ^1^H NMR spectra colour map of metabolite significance variations between the groups, (**D**) siR neg cont and siR HIF-1α, (**E**) siR neg cont and siR miR-210, (**F**) siR HIF-1α and siR miR-210. Peak in positive direction indicates the increased level of metabolite, peak in negative direction indicates decreased level of metabolite **(**X—undefined metabolite). Analysed by Bruker Topspin 3.1 and MestReNova 10.0 software (Mestrelab Research, Santiago de Compostela, Spain).

Predictive component analysis (PCA) score plots analysis was used to identify differences in metabolite representation between the analyzed siRNA negative control (siR neg cont) and siR HIF-1α groups, siR neg cont and siR miR-210, or siR HIF-1α and siR miR-210, respectively (Fig. 6A–C, Table 4). Identification of represented metabolites, their levels and loading plots were obtained from PCA analysis (Fig. 6D–F). In the analyzed groups we observed significant changes in the level of metabolites, their up- and down-regulation, see Table 5.

**Table 4:** ^1^HD NMR.

Number	Metabolite	Number	Metabolite	Number	Metabolite
1	Val, Ile, Leu	12	Choline	23	NAD^+^
2	Lactate	13	Carnitine	24	NADH
3	Ala	14	Myoinositol	25	ATP
4	DNA	15	Thr	26	AMP, adenine
5	Acetate	16	Glucose	27	Inosine
6	Glu	17	Ser	28	NADP^+^
7	Glutathione	18	Phe	29	Pro
8	Asp	19	Tyr	30	His
9	2-oxoglutharate	20	UDP-Na	31	Maltose
10	Lys	21	UDP-diphosphoglucuronate	32	GTP
11	Creatine/phosphocreatine	22	NADPH	33	malate

**Table 5:** Change in total value of metabolites in analysed groups (based on Table 4).

	Increase	Decrease
siR neg cont—HIF-1α	5, 6, 26, 29, 13	23, 9, 15, 17, 18, 19, 28
siR neg cont—miR-210	3, 7, 11, 12, 14, 18, 27	2, 5, 6, 9, 13, 16, 22, 24, 26
HIF-1α—miR-210	3, 14, 15, 18	6, 9, 10, 11, 13, 17, 18, 19, 25, 26, 27, 28, 29

PCA score plot of siR neg cont and siRNA HIF-1α cell A375 samples was obtained using one predictive and four orthogonal components, with PC1 of 59.02% and PC2 of 24.69%. OPLS-DA score plot of siR neg cont and siRNA miR-210 samples using one predictive and four orthogonal components, with PC1 of 64.89% and PC2 of 20.24%. And of HIF-1α and siRNA miR-210 cell A375 samples using one predictive and four orthogonal components, with PC1 of 56.56% and PC2 of 27.81%.

### Cell viability

**Figure 7:**
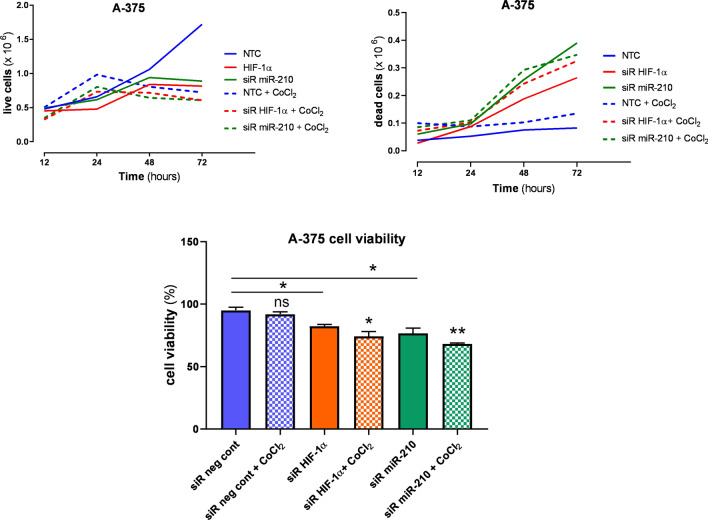
Cell growth line of each analyzed group per live and dead cells (12–72 h); cell viability (48 h).

Cell viability test by the Tryptan Blue experiment exhibited inhibition of cell proliferation for melanoma A375 cells after HIF-1α and miR-210 gene silencing but not for siR neg cont melanoma cells in normoxia nor hypoxia. Hypoxic conditions did not affect cell viability (Fig. 7). Our present study indentified a key miRNA similar to HIFs which inhibition could induce apoptosis or apoptosis-like cell death.

By analyzing the growth curves of the experimental groups, we can observe that A-375 cells affected by HIF-1α siRNA, and miR-210 siRNA had a significantly lower number of cells at 72 h compared to siR neg cont in normoxia as well as in hypoxia. By analogy, we detected a trend in the growth of "dead" cells. We observed a significant increase in the experimental group after silencing of the HIF-1α, and miR-210 genes compared to siR neg cont in both oxygen conditions. Gene silencing to siR neg cont significantly reduced the viability of A-375 melanoma cells in both normoxia and hypoxia. We detected an insignificant change in viability when comparing siR neg cont in normoxia and hypoxia (Fig. 7, supplementary data ST 7).

## Discussion

By suppressing the expression of miR-210 we wanted to introduce metabolic changes comparable with suppressing HIF-1α transcription factor similar to our previously reported study on malignant melanoma cell line SK-MEL-30^66^. Both conditions yielded reduction in the MITF-M expression and increase in the PDHA1 expression, which are reflected in the change of cell metabolism. It is well known that increased PDHA1 activity inhibits the Warburg effect and induces apoptosis^67^.

Our goal was to induce conditions that would minimize this effect. We detected slightly reduced MITF-M expression by HIF-1α gene silencing and a similar effect was observed by attenuating miR-210. We detected an increase in PDHA1 gene expression activity and at the same time a decrease in MITF-M expression activity by gene silencing of HIF-1α as well as miR-210. This effect elicited in regulatory gene pathways was reflected in mitochondrial activity and stimulation of pro-apoptotic signals. HIF-1α positively affects MITF-M expression under hypoxic conditions^48,68^. Increased activation of HIF-1α leads to MITF-M hyperactivation which negatively regulates PDHA1 expression^69^. As a siRNAs are large, polyaniomic molecules unstable in biological media and are capable of causing unwanted immune responses^70^ which can lead to ineffective reduced amount of target protein was used lipoid vector which increased diffusion of target siRNA into a cell because a naked siRNA only partialy passively diffuse through cellular membrane^71^. The low efficiency of siRNA HIF-1α transfection in melanoma cell line could be because of non-appropriate siRNA concentration, or the serum quality was not sufficient for target siRNA transfection, or the read-out time was not optimal for siRNA HIF-1α in melanoma cells but it was optimal time window for the rest of experimental condition. As we focused in our study on comparing the effect of gene silencing HIF-1α and miR-210 on the monitored processes (O_2_^•−^ production, accumulation of NADH in the cytosol, and the efficiency of mitochondria in ATP production) we think that the less successful gene silencing could have an effect on the preservation of pro-survival effect of the HIF-1α regulatory pathway.

From the general equation of mitochondria ROS generation NADH + 2 O_2_ → NAD^+^  + 2 O_2_^•−^  + H^+^^72^, an increased concentration of NADH leads to an increased concentration of O_2_^•−^. Non-physiological overexpression of HIF-1α reduces ROS levels^73^, thereby protecting the cell from oxidative stress-induced apoptosis, thus enhancing tumorigenesis. Some authors claim that the production of O_2_^•−^ could be reduced by suppressing NADH or by reducing the NADH/NAD^+^ ratio^74^. The decrease in the NADH/NAD^+^ ratio in the cytosol of cells reflects the change in the energy substrate preference by anaplerotic reactions of citric acid cycle^75,76^. Contrariwise, our experimental measurements show that the reduction of NADH accumulation, the NADH/NAD^+^ ratio, in the cytosol leads to a significantly increased production of O_2_^•−^ in normoxia. Mitochondria which effectively produce ATP have a lower NADH/NAD^+^ ratio and their production of ROS depends on local concentration of oxygen, mitochondrial membrane potencial, and the CoQH_2_/CoQ ratio in addition to the NADH/NAD^+^ ratio^77^. A relatively low level of the NADH/NAD^+^ ratio is associated with a low level of the CoQH_2_/CoQ ratio and leads to an increase in ROS production through respiratory complex III^78^. We detected minimal, essentially no change in NADH levels, which again resulted in an increased ROS production in hypoxia, while the increase in the ratio of free NADH/NAD^+^ in hypoxic conditions reflects the so-called glycolytic metabolism of malignant cells that behave according to Warburg's principles.

All gene manipulations such as gene activation and gene silencing can involve cytotoxicity, but to the best of our knowledge there is no described cytotoxic affect of miR-210 gene silencing. We hypothesize that the observed conditions such as a decrease in the level of the NADH/NAD^+^ ratio and an increase in the ROS level are caused by reactivation of the impaired mitochondria metabolism and apoptic processes.

Control cells showed minimal mitochondrial activity in both normoxia and hypoxia^79^, which again corresponds to Warburg's definition^80^. Restriction of the gene activity of hypoxic factors HIF-1α as well as miR-210 increased mitochondrial viability in both normoxic and hypoxic conditions. We observed a significant increase in ATP-linked respiration, H^+^-leak, and reverse capacity. Increased OXPHOS mediated by HIFs attenuation leads to increased mitochondrial biogenesis^81,82^. An increase in OXPHOS may also be a manifestation of cell adaptation to the conditions that have arisen, leading to de novo resistance of melanoma cells to inhibitors of the MAPK pathway and subsequently to oxidative stress, so-called combination of inhibition of the MAPK pathway and OXPHOS^37^.

OCR measurements indicate that the significance of silencing of hypoxic factor miR-210 gene products has a similar effect on the activation of respiratory complexes as attenuation of hypoxic factor HIF-1α gene products. The effect of gene silencing is broader in controlling expression of a number of other tumorigenic transcription and growth factors which also belong to miR-210. We also detected a significant increase in basal glycolysis after both the HIF-1α gene and miR-210 silencing. The proportion of non-glycolytic acidification after silencing of HIF-1α in normo- and hypoxia and miR-210 in normoxia was significant, which may reflect an increased production of acidic metabolites.

Some studies claim that suppressing the expression of hypoxic transcription factors will decrease ROS production^19,83,84^, which we did not observe in our experiments. This atypical trend of increasing ROS activity was shown also by Zhao et al. who reported that inhibitors of HIF-1 inhibit reprogramming of cancer cell from OXPHOS to anaerobic glycolysis, increase intratumoral ROS production, and eventually supress the formation of metastatic tumours^85^. Moreover, the inhibition of miR-210 and its target proteins such as ISCU1/2 also increases ROS production^86^. The downregulation of HIFs and hypoxa-miR can upregulate ROS production by a positive/negative feedback loop^87^. The levels of ROS widely fluctuate during cell life because ROS in cancer cells can increase and decrease based on variations in the expression of SOD in cancer cells^88,89^. This fact can have a large effect on the measured ROS production.

ROS activate various cellular signalling pathways as PI3K/Akt, MAPK, NF-κB, and p53 which play an important role in cell survival and death caused by apoptosis^90^. In addition to the increase in specific gene and protein products, apoptosis can be defined by the presence of characteristic metabolites used to activate apoptotic processes, or the products of an apoptotic process which we uniformly refer to as "find-me" signals^91–94^.

NMR spectra of the metabolites in cells affected by siR HIF-1α and siR miR-210 show significant changes in the representation of apoptotic signals defined by an increased concentration of amino acids (Ala, Phe, Ser, Glu, etc.) due to the breakdown of cellular organelles, an increase in nucleotides as a manifestation of the breakdown of the cell nucleus, and an increase in carnitine concentration (in some cases it has a protective role against apoptosis/necrosis especially in muscle cells during hard work) which can serve as a stimulant of the apoptotic process in cancer cells, an increase in myo-inositol, UDP-derivates, and alanine^95,96^ compared to siR neg cont cells. Detected increase in mitochondrial activity (using the Seahorse Assay) which led to the accumulation of ROS (analysed by MitoSox) and thus to apoptosis of cells was reflected in an overall decrease in ATP in the cells affected by siRNAs detected by NMR. We also observed an increase in NAD(P)^+^ and a decrease in NADH in the NMR spectra of cells affected by gene silencing compared to siR neg cont. This NMR spectral data confirms the measurements obtained with the Peredox m-Cherry Assay, where we similarly observed a decrease in free NADH and an increase in NAD^+^.

In summary, our work shows a direct link between the induction of apoptosis and the elimination of the effect of hypoxic transcription factors in induced pseudohypoxia in MM A375 cells by post-transcriptional repression of the HIF-1α and miR-210 gene. We observed a significant decrease in the accumulation of free NADH in the cytosol of cells, and an increase in OCR in mitochondrial activity. This phenomenon may be due to the activation of respiratory complexes and thus the use of OXPHOS to generate energy, making more efficient use of cytosolic glycolytic metabolism. ECAR measurements show that glycolysis increased only after HIF-1α attenuation. After inactivation of miR-210, we observed a decrease in glycolytic activity, but an increase in ATP production by the OXPHOS pathway. Activation of respiratory complexes also led to an increase in O_2_^•−^ production and the flushing of "find-me" apoptotic signals. The growth curve data shows that the cells exhibit an increase of apoptotic activity after gene silencing of both HIF-1α and miR-210. It can be concluded that affected malignant melanoma cells undergo apoptosis or process “death-like” apoptosis by reactivating mitochondrial metabolism.

These findings could be used in the future to develop drugs that target expression of miR-210 (as our data shows a thin connection between miR-210 and HIF-1α on mitochondria respiratory complexes). The treatment would focus predominantly on those cells that, due to elevated levels of miR-210, detach mitochondrial metabolism from cellular energy production.

## Material and methods

### Malignant melanoma cell culture

Amelanotic malignant melanoma cell line A375 (Cell Bank Graz, Medical University of Graz, Austria) was cultured in Dulbecco's Modified Eagle's Medium (DMEM) with 25 mM glucose, 2 mM glutamine, 10% Fetal Calf Serum (FCS), 100 U/ml penicillin, 0.1 mg/ml streptomycin and 1.25 µg/ml amphotericin in a thermoincubator at 37 °C and 95% O_2_, 5% CO_2_ atmosphere.

We stimulated the pseudohypoxic environment by adding 100 µM CoCl_2_ · 6H_2_O to the culture medium for 24 h after siRNA transfection. Cobalt(II) chloride hexahydrate is a chemical inducer of HIF-1,2,3^95^ and acts by three different stabilization mechanisms of HIF-1α:

1. CoCl_2_ stabilizes HIF-1α through antagonism with Fe^2+^ which is an essential cofactor along with O_2_ for PHD (prolylhydroxylase) which degrades HIF-1α;
2. partial inhibition of PHD and depletion of ascorbate leading to maintenance of HIF-PHD and HIF inhibitory factor (FIH) whereby HIF remains in active form;
3. direct binding of cobalt to HIF-1α, which can protect it from degradation via the VHL-dependent and VHL-independent pathways^96^.

It has also been documented that CoCl_2_ is involved in the selective activation of HIF-1α signaling^96^.

### Gene silencing and geneticaly encoded biosensores

Gene silencing of HIF-1α and miR-210 was induced overnight (12 h) by adenoviral transfection (100 nM of the appropriate siRNA with 2.5 μg/ml TransFast transfection reagent (Promega, Madison, WI, USA)) HIF-1α siRNA in encoded sequence 5'-CCA CCA CUG AUG AAU UAA AUU TT-3 '(Microsynth, Oligo ID # 2,808,643), and siRNA miR-210 in the coding sequence 5'-UCA GCC GCU GUC ACA CGC ACA GTT-3' (Microsynth, Oligo ID # 2,808,645), and negative siRNA control (Thermofisher, # 4,390,843) on A375 malignant melanoma subconfluent cells. The genetically encoded biosensors Peredox-mCherry, and MitoTracker Red CMXRos were transfected with the appropriate plasmid DNA Peredox-mCherry (pcDNA3.1), and MitoTracker Red CMXRos (tetramethylrhodamine methyl ester and 10-N-nonyl acridine orange) into A375 subconfluent cells. Cells which were treated with negative siRNA control (siR neg cont) were used as control. The cells were used for analysis after 48 h of transfection.

### mRNA/miRNA isolation and RT-PCR

The peqGOLD total RNA kit (Peqlab; Erlangen, Germany, cat. No.: 12–6834-02) was used to isolate total RNA and miRNA. Isolated nucleic acids were transcribed into cDNA using a cDNA synthesis kit (Applied Biosystems; Foster City, CA) and a thermocycler (Peqlab). RT-PCR amplification was performed using a QuantiFast SYBR Green RT-PCR kit (Qiagen; Hilden, Germany) and LightCycler 480 (Roche Diagnostics; Vienna, Austria). The obtained data were analyzed using REST Software (Qiagen). Relative gene expression was normalized to the housekeeping gene GAPDH, and U6 respectively. The primer sequences are listed in supplementary data (ST 1).

The total number of mRNA molecules varies with cell size, the number of cells, cell metabolic status and cell cycle phase^97^ and thus results in microenvironment higher or lower gene expression, even under gene silencing. The relevancy or effectivity of gene silencing can be „measured” as synergistically up/downregulation of linked genes^98,99^.

### Peredox-mCherry T-Sapphire assay

The accumulated NADH/NAD^+^ ratio was determined with the genetically encoded Peredox-mCherry T-Sapphire (CFP/YFP) fluorescent biosensor (1.5 μg/ml plasmid with 2.5 μg/ml TransFast transfection reagent (Promega, Madison, WI, USA)) whose change in fluorescence intensity after NADH molecule binding was detected by Real Time Single Cell Imaging Microscopy LSM Pascal (Zeiss, Germany) with 40×–100× oil immersion objective and optical distance < 1 µm with laser excitation of the first wavelength 430 nm and the second excitation wavelength 570 nm, the excitation time for channel 1 was 200 ms, and for channel 2 was 50 ms. The method is based on a sensitive fluorescent biosensor for changing the NADH/NAD^+^ ratio combining circulating GFP T-Sapphire with bacterial NADH-binding protein Rex whose fluorescence intensity increases only by reversible binding of NADH to the complex^100,101^. A375 cells were stimulated to produce and consume NADH by adding 500 mM lactate, or 500 mM pyruvate to the extracellular Na^+^/Ca^2+^ solution (121.5 mM NaCl, 25 mM NaHCO_3_, 2.5 mM KCl, 2 mM CaCl_2_, 1. 25 mM NaH_2_PO_4_, 1 mM MgCl_2_). We performed measurements in triplicate for each monitored condition.

### MitoTracker Red CMXRos assay

MitoTracker Red CMXRos is a passively diffusing permeable probe containing mild thiol-reactive chloromethyl that accumulates in active mitochondria. The determined intensity of red fluorescence produced by O_2_^•−^ by cells incubated with the MitoTracker Red CMXRos biosensor (200 nM MitoTracker Red CMXRos) in the dark for 10 min before the actual cell measurement. We detected the changes in ROS using confocal spinning disk microscope (Axio Observer.Z1 from Zeiss, Gottingen, Germany) equipped with 100 × objective lens (Plan-Fluor × 100/1.45 Oil, Zeiss), a motorized filter wheel (CSUX1FW, Yokogawa Electric Corporation, Tokyo, Japan) on the emission side, AOTF-based laser merge module for laser line 405, 445, 473, 488, 515, and 561 nm (Visitron Systems), and a Nipkow-based confocal scanning unit (CSUX1, Yokogawa Electric Corporation). Cells A375 with MitoTracker Red CMXRos were alternately excited with 579 nm laser lines, and emissions were acquired at 599 nm using a charged CCD camera (Cool SNAP-HQ, Photometrics, Tucson, AZ, USA). Z-stacks of channel in 0.2 µm increments were recorded. The VisiView acquisition software (Universal Imaging, Visitron Systems) was used to acquire the imaging data. Modified protocol according to Madreiter-Sokolowski et al.^102^.

### Seahorse assay

The mitochondrial activity of A375 cells was detected using the Seahorse Assay. Cells were measured at 100% confluence in Cell-Tak covered by XF96—96 well polystyrene cell culture microplates (Seahorse Bioscience, Agilent; California, US). We determined the OCR (oxygen consumption rate) and ECAR (extracellular acidification rate) using an XF96 extracellular flow analyzer. The values of OCR and ECAR were measured every 7 min. After 15 min. basal measurements were injected with solutions of 2 μM Oligomycin followed by 2.5 μM Antimycin. Therefore, we analyzed the OCR data, and the ECAR data was according to the Seahorse XF96 protocol. We performed measurements in triplicate for each monitored condition.

### 1D ^1^H NMR

All ^1^H ^1^D NMR experiments were performed at 310 K on a Bruker Avance III 500 MHz spectrophotometer equipped with a TXI probe (Bruker Daltonics, Bremen, Germany). A 1D CPMG pulse sequencer (Carr-Purcell-Meiboom-Gill) with cpmgpr1d parameters, 73,728 points in F1, 12,019.230 Hz spectral width, 2048 pass, with a cyclic delay of 4 secs, was used for the measurement, with water suppression using presaturation. We used the reference chemical database Madison-Qingdao Metabolomics Consortium Database to analyze the obtained metabolites^103^ and all determined metabolites were compared with reference compounds. We acquired, processed, and evaluated the results using Bruker Topspin 3.1, and MestReNova 10.0 software (Mestrelab Research, Santiago de Compostela, Spain). We related the concentration of metabolites to the TSP standard. We performed measurements in triplicate for each monitored condition.

### Cell viability

The proliferative capacity and viability of A375 malignant melanoma cells was detected using the Trypan Blue Staining Assay. Cells were seeded at the density of 150,000 per well in a 24-well plate and cultured for 72 h. Cells were transfected with the appropriate siRNA before starting cell proliferation measurements. The percentage of viable cells (at 48 h) was calculated according to Sigma-aldrich recommendations^104^.

### Statistical analysis

The presented experimental data were evaluated using GraphPad Prism 5.04 and represent the mean values ± SEM of three independent measurements. Multiple t-test, one-way, and two-way ANOVA was used (Tukey's Multiple Comparison test) for statistical analysis. Statistically significant results were found to have a *P* value < 0.01 is statistically significant, a *P* value < 0.001 highly significant, a *P* value < 0.0001 strongly significant^105^.

## Supplementary Information

Supplementary Information.
