# Supplementary material for: Effect of hypoxia factors gene silencing on ROS production and metabolic status of A375 malignant melanoma cells
Source: Sci Rep. 2021 May 14;11:10325. doi: 10.1038/s41598-021-89792-2 (PMC8121821; doi:10.1038/s41598-021-89792-2)
Supplement: Supplementary file 1 — Supplementary Information. [file 41598_2021_89792_MOESM1_ESM.docx]

**Effect of hypoxia factors gene silencing on ROS production and metabolic status of A375 malignant melanoma cells**

(Full-length Article)

Ivana Špaková^1^, Miroslava Rabajdová^1*^, Helena Mičková^2^, Wolfgang F. Graier^3,4^, Mária Mareková^1^

^1^Department of Medical and Clinical Biochemistry, Pavol Jozef Šafárik University in Košice, Faculty of Medicine, Slovakia; ^2^Department of Biology, Pavol Jozef Šafárik University in Košice, Faculty of Medicine, Slovakia; Gottfried Schatz Research Center for Cell Signaling, Metabolism and Aging Molecular Biology and Biochemistry, Medical University of Graz, Graz, Austria; ^4^BioTechMed, Graz, Austria

Corresponding author:

Assoc. prof. Dr. Miroslava Rabajdová, PhD.

[miroslava.rabajdova@upjs.sk](mailto:miroslava.rabajdova@upjs.sk)

Department of Medical snd Clinical Biochemistry

Faculty of Medicine, Pavol Jozef Šafárik University in Košice

Trieda SNP 1, 04011 Košice, Slovakia

**Supplementary data**

| **gene** | **forward (sense) sequence** | **reverse (antisense) sequence** | **ID #** |
| --- | --- | --- | --- |
| GAPDH | TGGGGCCAAAAGCATCATCTC | GCCGCCTGCTTCACCACCTTCTT |  |
| MITF-M | TAAACTCCCCGCGCTG | CTTCCACTGCTGGAAAGTGA | R0920B03/B04 |
| HIF-1α | CGTTCCTTCGATCAGTTGTC | TCAGTGGTGGCAGTGGTAGT | L6237B06/B07 |
| PDHA1 | ATGTGGAAGTGAGGAAGGAG | TCGCTGGAGTAGATGTGGTA | R0443A07/A08 |
| miR-210 | CTGTGCGTGTGACAG | GTGCAGGGTCCGAGGT | L6237B02/B03 |
| U6 | CTCGCTTCGGCAGCACA | AACGCTTCACG AATTTGCGT | L6237B04/B05 |

**ST 1 –** F/R sequences of primers for RT- PCR were obtained from Invitrogen (Vienna, Austria).

| Multiple t-test normoxia vs. hypoxia for each gene | | | | | | | | | |
| --- | --- | --- | --- | --- | --- | --- | --- | --- | --- |
|  |  | | **P value** | |  |  | | | **P value** |
| **MITF-M** | *NTC* | | 0.4411 | | **PDHA1** | *NTC* | | | 0.7956 |
|  | *siR HIF-1α* | | 0.5533 | |  | *siR HIF-1α* | | | 0.4276 |
|  | *siR miR-210* | | 0.3975 | |  | *siR miR-210* | | | 0.7944 |
|  | | | | | | | | | |
| **HIF-1α** | *NTC* | | 0.3181 | | **miR-210** | *NTC* | | | 0.9449 |
|  | *siR HIF-1α* | | 0.3359 | |  | *siR HIF-1α* | | | 0.6908 |
|  | *siR miR-210* | | 0.8124 | |  | *siR miR-210* | | | 0.6437 |
| Multiple t-test | | | | | | | | | |
|  | | **normoxia** | | **P value** | | | **hypoxia** | **P value** | |
| **MITF-M vs. HIF-1α** | | *NTC* | | - | | | *NTC* | 0.9151 | |
|  | | *siR HIF-1α* | | 0.6137 | | | *siR HIF-1α* | 0.2465 | |
|  | | *siR miR-210* | | 0.3095 | | | *siR miR-210* | 0.5227 | |
|  | | | | | | | | | |
| **MITF-M vs. PDHA1** | | **normoxia** | | **P value** | | | **hypoxia** | **P value** | |
|  | | *NTC* | | - | | | *NTC* | 0.8249 | |
|  | | *siR HIF-1α* | | 0.7485 | | | *siR HIF-1α* | 0.3023 | |
|  | | *siR miR-210* | | 0.2025 | | | *siR miR-210* | 0.5771 | |
|  | | | | | | | | | |
| **HIF-1a vs. PDHA1** | | **normoxia** | | **P value** | | | **hypoxia** | **P value** | |
|  | | *NTC* | | - | | | *NTC* | 0.7604 | |
|  | | *siR HIF-1α* | | 0.9241 | | | *siR HIF-1α* | 0.0315 | |
|  | | *siR miR-210* | | 0.0049 | | | *siR miR-210* | 0.9637 | |
|  | | | | | | | | | |
| **HIF-1a vs. miR-210** | | **normoxia** | | **P value** | | | **hypoxia** | **P value** | |
|  | | *NTC* | | - | | | *NTC* | 0.3181 | |
|  | | *siR HIF-1α* | | 0.5233 | | | *siR HIF-1α* | 0.9750 | |
|  | | *siR miR-210* | | 0.1310 | | | *siR miR-210* | 0.8051 | |
|  | | | | | | | | | |
| **MITF-M vs. miR-210** | | **normoxia** | | **P value** | | | **hypoxia** | **P value** | |
|  | | *NTC* | | - | | | *NTC* | 0.6643 | |
|  | | *siR HIF-1α* | | 0.3133 | | | *siR HIF-1α* | 0.3105 | |
|  | | *siR miR-210* | | 0.9604 | | | *siR miR-210* | 0.3079 | |
|  | | | | | | | | | |
| **PDHA1 vs. miR-210** | | **normoxia** | | **P value** | | | **hypoxia** | **P value** | |
|  | | *NTC* | | - | | | *NTC* | 0.8765 | |
|  | | *siR HIF-1α* | | 0.5109 | | | *siR HIF-1α* | 0.5110 | |
|  | | *siR miR-210* | | 0.0768 | | | *siR miR-210* | 0.9639 | |

**ST 2 –** statistics of relative gene expression

| Unpaired T-test | | | | |
| --- | --- | --- | --- | --- |
|  | **normoxia** | **P value** | **hypoxia** | **P value** |
| **to NTC** | *NTC* | - | *NTC* | 0.1522 |
|  | *siR HIF-1α* | 0.0031 | *siR HIF-1α* | 0.0439 |
|  | *siR miR-210* | <0.0001 | *siR miR-210* | 0.2501 |
| **to NTC + CoCl_2_** | *NTC* | 0.1522 | *NTC* | - |
|  | *siR HIF-1α* | <0.0001 | *siR HIF-1α* | 0.1122 |
|  | *siR miR-210* | <0.0001 | *siR miR-210* | 0.7098 |
| Ordinary one-way ANOVA Tukey’s multiple comparisons test | | | | |
|  | **normoxia** | **P value** | **hypoxia** | **P value** |
|  | *NTC vs. siR HIF-1α* | 0.0057 | *NTC vs. siR HIF-1α* | 0.3470 |
|  | *NTC vs. siR miR-210* | <0.0001 | *NTC vs. siR miR-210* | 0.9147 |
|  | *HIF-1α vs. miR-210* | 0.5656 | *HIF-1α vs. miR-210* | 0.6665 |

**ST 3 –** statistics of NADH/NAD^+^ ratio

| Unpaired T-test | | | |
| --- | --- | --- | --- |
| ***Basal respiration*** | | | |
| **to NTC** | **P value** | **k NTC + CoCl_2_** | **P value** |
| *NTC + CoCl_2_* | 0.0010 | *NTC* | - |
| *siR HIF-1α* | <0.0001 | *siR HIF-1α + CoCl_2_* | <0.0001 |
| *siR miR-210* | <0.0001 | *siR miR-210 + CoCl_2_* | <0.0001 |
| ***ATP-linked respiration*** | | | |
| **to NTC** | **P value** | **k NTC + CoCl_2_** | **P value** |
| *NTC + CoCl_2_* | 0.7773 | *NTC* | - |
| *siR HIF-1α* | 0.0263 | *siR HIF-1α + CoCl_2_* | 0.1066 |
| *siR miR-210* | 0.0271 | *siR miR-210 + CoCl_2_* | <0.0001 |
| ***Mitochondrial reverse capacity*** | | | |
| **to NTC** | **P value** | **k NTC + CoCl_2_** | **P value** |
| *NTC + CoCl_2_* | 0.0033 | *NTC* | - |
| *siR HIF-1α* | <0.0001 | *siR HIF-1α + CoCl_2_* | <0.0001 |
| *siR miR-210* | 0.0010 | *siR miR-210 + CoCl_2_* | <0.0001 |
| ***H^+^ leak*** | | | |
| **to NTC** | **P value** | **k NTC + CoCl_2_** | **P value** |
| *NTC + CoCl_2_* | 0.0422 | *NTC* | - |
| *siR HIF-1α* | 0.2538 | *siR HIF-1α + CoCl_2_* | 0.0107 |
| *siR miR-210* | 0.0153 | *siR miR-210 + CoCl_2_* | 0.0023 |
| ***Non-mitochondrial respiration*** | | | |
| **to NTC** | **P value** | **k NTC + CoCl_2_** | **P value** |
| *NTC + CoCl_2_* | 0.0067 | *NTC* | - |
| *siR HIF-1α* | 0.0056 | *siR HIF-1α + CoCl_2_* | 0.0005 |
| *siR miR-210* | 0.0002 | *siR miR-210 + CoCl_2_* | 0.0059 |
| ***Reverse capacity*** | | | |
| **to NTC** | **P value** | **k NTC + CoCl_2_** | **P value** |
| *NTC + CoCl_2_* | 0.5928 | *NTC* | - |
| *siR HIF-1α* | 0.0026 | *siR HIF-1α + CoCl_2_* | 0.0224 |
| *siR miR-210* | 0.0290 | *siR miR-210 + CoCl_2_* | 0.0117 |

**ST 4 –** statistics of OCR values

| Unpaired T-test | | | |
| --- | --- | --- | --- |
| ***Glycolysis*** | | | |
| **to NTC** | **P value** | **k NTC + CoCl_2_** | **P value** |
| *NTC + CoCl_2_* | 0.0011 | *NTC* | - |
| *siR HIF-1α* | 0.0089 | *siR HIF-1α + CoCl_2_* | <0.0001 |
| *siR miR-210* | 0.0080 | *siR miR-210 + CoCl_2_* | 0.0075 |
| ***Maximum of glycolytic capacity*** | | | |
| **to NTC** | **P value** | **k NTC + CoCl_2_** | **P value** |
| *NTC + CoCl_2_* | 0.0029 | *NTC* | - |
| *siR HIF-1α* | 0.4312 | *siR HIF-1α + CoCl_2_* | 0.0069 |
| *siR miR-210* | 0.1582 | *siR miR-210 + CoCl_2_* | 0.8899 |
| ***Glycolytic reserve*** | | | |
| **to NTC** | **P value** | **k NTC + CoCl_2_** | **P value** |
| *NTC + CoCl_2_* | 0.0186 | *NTC* | - |
| *siR HIF-1α* | 0.0237 | *siR HIF-1α + CoCl_2_* | 0.1749 |
| *siR miR-210* | 0.0203 | *siR miR-210 + CoCl_2_* | 0.0530 |

**ST 5 –** statistics of ECAR values

| Unpaired T-test | | | | |
| --- | --- | --- | --- | --- |
|  | **normoxia** | **P value** | **hypoxia** | **P value** |
| **to NTC** | *NTC* | - | *NTC* | <0.0001 |
|  | *siR HIF-1α* | <0.0001 | *siR HIF-1α* | <0.0001 |
|  | *siR miR-210* | <0.0001 | *siR miR-210* | <0.0001 |
| **to NTC + CoCl_2_** | *NTC* | <0.0001 | *NTC* | - |
|  | *siR HIF-1α* | <0.0001 | *siR HIF-1α* | <0.0001 |
|  | *siR miR-210* | <0.0001 | *siR miR-210* | 0.3133 |
| ANOVA summary | | | | |
|  | **normoxia** | **P value** | **hypoxia** | **P value** |
|  | *NTC* | <0.0001 | *NTC* | <0.0001 |
|  | *siR HIF-1α* |  | *siR HIF-1α* |  |
|  | *siR miR-210* |  | *siR miR-210* |  |

**ST 6 –** statistics of MitoTracker Red CMXRos values

| Unpaired T-test | | | |
| --- | --- | --- | --- |
| **to NTC** | **P value** | **to NTC + CoCl_2_** | **P value** |
| *NTC + CoCl_2_* | 0,2766 | *NTC* | - |
| *siR HIF-1α* | 0,0233 | *siR HIF-1α + CoCl_2_* | 0,0274 |
| *siR miR-210* | 0,0335 | *siR miR-210 + CoCl_2_* | 0,0038 |

**ST 7 –** statistics of the cell viability (in 48 hrs)
